# *Nicotiana benthamiana* as a model system for dissecting plant-pathogen interactions

**DOI:** 10.3389/fpls.2026.1770827

**Published:** 2026-03-03

**Authors:** Zhengchao Yang, Weijie Xu

**Affiliations:** 1 College of Life and Environmental Sciences, Hangzhou Normal University, Hangzhou, China; 2 College of Forestry and Biotechnology, Zhejiang Agricultural and Forestry University, Hangzhou, China

**Keywords:** effector-triggered immunity (ETI), Nicotiana benthamiana, pattern-triggeredimmunity (PTI), plant-pathogen interactions, RDR1

## Abstract

*Nicotiana benthamiana* is widely used as a model plant for studying plant-pathogen interactions owing to its genetic background and experimental accessibility. Its allopolyploid genome, high susceptibility to diverse pathogens, and distinct composition of RNA silencing-related genes provide a suitable system for investigating host responses during infection. Studies in *N.benthamiana* have enabled detailed analyses of interactions with viruses, viroids, bacteria, fungi, and oomycetes, and have contributed to the characterization of antiviral RNA silencing, pattern-triggered immunity (PTI), effector-triggered immunity (ETI), and MAPK-mediated signaling pathways. Premature transcriptional termination resulting in a nonfunctional *NbRDR1* constitutes a key factor influencing antiviral RNA silencing regulation and host–virus interactions in this species. *N.benthamiana* has also been used to dissect immune receptor-mediated signaling and downstream defense responses to non-viral pathogens, including reactive oxygen species production and programmed cell death. In addition, the availability of virus-induced gene silencing and *Agrobacterium*-mediated transient expression systems has facilitated functional analyses and comparative studies across plant species. This review integrates molecular insights from diverse pathosystems and evaluates limitations relevant to interpreting results obtained in *N.benthamiana*.

## 1 Introduction

**Figure 1:**
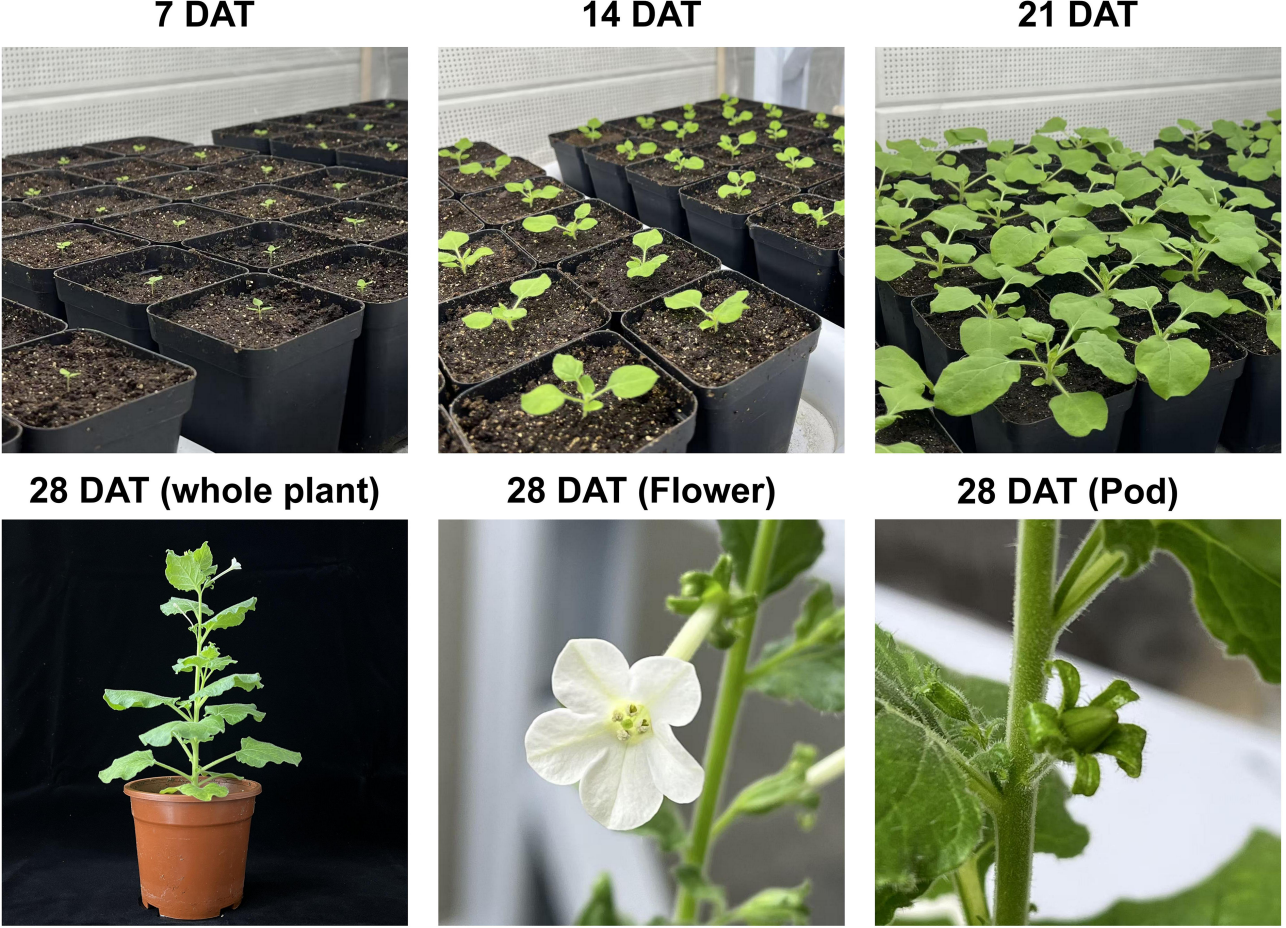
Growth and developmental progression of plants at different days after transplanting (DAT) under greenhouse conditions. Representative images show plant morphology at 7, 14, and 21 DAT during early vegetative growth. At 28 DAT, images illustrate the whole plant, as well as reproductive organs including flowers and pods.

The genus Nicotiana originated in South America and subsequently spread to multiple continents, including Australia and the South Pacific (Goodspeed, 1947). *Nicotiana benthamiana* (*N.benthamiana*), a native Australian species, diverged from its ancestral lineages approximately 20 million years ago and successfully adapted to dramatic climatic changes that transformed large parts of the Australian continent into arid regions (Goodspeed, 1947). These evolutionary processes resulted in a species with distinct genetic and physiological characteristics (**Figure 1**). These features later proved advantageous for experimental research (Baulcombe, 2004).

*N.benthamiana* belongs to the Solanaceae and is a heterotetraploid species formed through ancient hybridization events, and has a genome with 19 chromosomes (2n=19). Comparative genomic analyses have revealed that its genome originated from hybridization between ancestral lineages related to *Nicotiana glauca* and *Nicotiana sylvestris*, with subsequent polyploidization contributing to genetic redundancy and functional diversification (Kelly et al., 2013**;** Schiavinato et al., 2020). Such genomic features are thought to enhance phenotypic plasticity and may partially explain the exceptional adaptability and experimental responsiveness of *N.benthamiana* (Wang et al., 2024).

Beyond its evolutionary background, *N.benthamiana* has become a premier model plant due to a unique combination of experimental advantages. It is easy to cultivate, generates abundant biomass, and exhibits well-characterized patterns of gene expression regulation and post-translational modification (Bally et al., 2018). Importantly, *N.benthamiana* is highly susceptible to a broad range of plant viruses and supports an efficient *Agrobacterium*-mediated transient expression system. These properties enable rapid functional analyses of gene regulation, protein-protein interactions, and subcellular localization.

The exceptional sensitivity of *N.benthamiana* to viral infection, coupled with its amenability to transient and stable genetic manipulation, has positioned it as a central model for dissecting plant-pathogen interactions. In this review, we summarize recent mechanistic studies investigating interactions between *N.benthamiana* and diverse pathogenic microorganisms, with a particular focus on immune signaling and pathogen virulence strategies. This synthesis provides a framework for future applications of *N.benthamiana* in plant disease resistance research and its development as a plant-based biotechnological platform.

## 2 RNA virus-host interactions studied in *N.benthamiana*

Throughout evolutionary history, interactions between plants and pathogens have been sharped by continuous selective pressures, with plant-virus interactions representing a particularly dynamic antagonistic relationship. RNA silencing is an important immune mechanism for plant defense against aberrant expression of exogenous RNAs, and the initiation of this mechanism in plants is largely dependent on the *RDRPs* gene family (Baulcombe, 2004). In *N.benthamiana*, the initiation of RNA silencing is usually associated with the *RDR1* and *RDR6* genes, and *NbRDR1* exhibits symptoms of susceptibility to viral infestation due to the insertion of a 72-bp fragment, e.g., high susceptibility to *tobacco mosaic virus* (TMV). The Arabidopsis *RDR6* (*AtRDR6*) homolog *NbRDR6*, on the other hand, is very conserved and is involved in responding to viruses such as *cucumber mosaic virus* (CMV) and *potato X virus* (PVX). In addition, *RDR6* is involved in short-range intercellular RNA silencing in *N.benthamiana***(**Qin et al., 2012).

In research of *N.benthamiana*’s interactions with viruses, despite the fact that both the *RDR1* and *RDR6* genes play important roles in plant RNA silencing, there are significant differences in the way their genes are expressed. *RDR1* is inducibly expressed and is only expressed in response to inducers such as salicylic acid (SA) or jasmonic acid (JA) (Pandey and Baldwin, 2007**;** Xu et al., 2003). Yang (Yang et al., 2004) demonstrated that *N.benthamiana* carries a naturally mutated, nonfunctional *RDR1* allele (*NbRDR1m*). Although *NbRDR1m* transcription is induced by salicylic acid following TMV infection, a premature stop codon prevents the production of a full-length functional protein. As a result, *NbRDR1m* fails to restrict viral accumulation and does not effectively limit disease progression.The conclusion that *NbRDR1* is not functional or has reduced activity is supported by the fact that transgenic plants inoculated with TMV showed improved lethality by transgenic transfer of *Medicago truncatula RDR1* gene (*MtRDR1*) into *N.benthamiana* (Lee et al., 2016), and that TMV infestation or SA induction can elicit *NtRDR1* activity in *N.tabacum*, and the accumulation of viral RNA was significantly higher than that of the wild type when the *RDR1*-deficient mutant *N.tabacum* was infested with TMV, but the viral proliferation was inhibited and the viral RNA content decreased in the SA-treated *RDR1*-deficient *N.tabacum* (Xie et al., 2001). These results suggest that SA possesses both an *RDR1* dependent and an independent defense pathway.

*RDR6* is constitutively expressed and is involved in RNA silencing independently of spatiotemporal specificity. *NbRDR6* removes viruses from root tip growth point meristematic tissues and inhibits the systemic spread of PVX (Schwach et al., 2005). It has been noted that *RDR1* and *RDR6* are also not exactly two independent RNA silencing initiation pathways. The introduction of *NtRDR1* into *N.benthamiana* by transgenic means was intended to compensate for its immunodeficiency. However, this resulted in the suppression of *NbRDR6*-dependent post-transcriptional gene silencing. The phenotype resembled that observed following *NbRDR6* gene silencing.(Ying et al., 2010), which suggests that *RDR1* has the function of suppressing *RDR6*-mediated antiviral RNA silencing. *RDR1* is mainly involved in plant defense against viruses, whereas *RDR6* has the same ability to defend against viruses and is also involved in the regulation of adversity response and growth and development (Qu et al., 2005**;** Peragine et al., 2004**;** Yang et al., 2011). Interestingly, according to Ying’s research, transfection of the active *RDR1* gene into *N.benthamiana* surprisingly increased the susceptibility of tobacco viruses other than TMV, *Turnip vein-clearing virus* (TVCV), and *Sunn hemp mosaic virus* (SHMV), such as *Plum pox potyvirus* (PPV), CMV, PVX, and *potato Y virus* (PVY), which explains the inhibitory effect of *RDR*1 expression on the function of *RDR6* (Ying et al., 2010). These observations suggest that the high viral sensitivity of *N.benthamiana* may have imposed strong selective pressure, favoring natural mutation of *NbRDR1* to maintain broad-spectrum antiviral defense mediated by *NbRDR6*. Moreover, the transition of central Australia from humid to arid conditions during *N.benthamiana* evolution may have further selected for *NbRDR1* inactivation, thereby enhancing *NbRDR6*-dependent stress and virus resistance and facilitating adaptation to climate change.

Dependence on RDR1 and RDR6 in plants can produce many dsRNAs, which subsequently undergo Dicer-Like (DCL) cleavage to form sRNAs (including siRNAs, miRNAs) induced with Argonaute (AGO) proteins to form the RNA-induced silencing complex (RISC), which can block expression of viral complementary RNAs by targeting the viral complementary RNAs to degrade or translationally repress them thereby blocking expression (Szittya and Burgyán, 2013**;** MaChado et al., 2017**;** Ma and Zhang, 2018**;** Yang and Li, 2018**;** Iwakawa and Tomari, 2022).

In *A.thaliana*, AGOs 1, 2, 4, 5, 7 and 10 are specific in virus defense (Carbonell and Carrington, 2015), and *N.benthamiana* may be similar. It has been shown that both *NbAGO1* and *NbAGO4* genes are required for systemic silencing in *N.benthamiana* by means of Virus-Induced Gene Sliencing (VIGS) (Jones and Dangl, 2006). Infection of *N.benthamiana* using *tomato ringspot virus* (ToRSV), followed by silencing of *NbAGO1*, prevents the recovery of necrotic symptoms in infected leaves with inhibition of viral translation, thus also demonstrating that the NbAGO1 protein is essential (Ghoshal and Sanfaçon, 2014). NbAGO2 has been frequently reported in studies, likely reflecting the central antiviral roles of AGO1 and AGO2 identified in *A.thaliana*. AGO1 acts as the primary defense by efficiently silencing exogenous RNA, whereas AGO2 serves as a secondary barrier that restricts viral accumulation. However, AGO1- and AGO2-mediated defenses are not fully simultaneous, as AGO2 activity is suppressed by miR403 when AGO1 is engaged (Harvey et al., 2011). AGO2-mediated antiviral defense is generally activated when AGO1-dependent primary defense is compromised, such as during infection by AGO1-suppressing viruses or artificial repression of AGO1. However, in *N.benthamiana*, repression of miR403, which negatively regulates *NbAGO2*, can activate NbAGO2 antiviral activity without disrupting AGO1 function. This challenges the prevailing view that AGO2 activation strictly depends on AGO1 inactivation.(Harvey et al., 2011**;** Diao et al., 2019). Scholthof utilized the VIGS technique using *Tobacco rattle virus* (TRV) as a vector to silence the expression of *NbAGO2* (Scholthof et al., 2011). When this gene expression was silenced, no significant effects on normal growth and development were observed in N. benthamiana; however, the plants displayed increased susceptibility after inoculation with *Tomato bushy stunt virus* (TBSV) (Scholthof et al., 2011). Following this, considering that by means of VIGS a virus would need to be introduced in the host, which could have an impact on the true phenotype by disrupting many of the host’s functions, the dsRNA hairpin was used instead to VIGS of *NbAGO2*, which resulted in a phenotype consistent with the previous one (Odokonyero et al., 2015). These two studies collectively demonstrate that NbAGO2 plays a facilitative role in antiviral defense. In addition to the above reports, there is still a great deal of enigma surrounding *NbAGOs* in *N.benthamiana*, and it remains to be further confirmed whether the remaining AGO proteins in *A.thaliana* that are more homologous to those in *N.benthamiana* also have specific antiviral effects in *N.benthamiana*.

*N.benthamiana* has four differently acting DCL proteins that each produce four distinct populations of small RNAs that exercise functions in subsequent binding to different AGO proteins. DCL2 and DCL4 are commonly thought to be associated with antiviral defense (Parent et al., 2015; Qin et al., 2017). A study in *N.benthamiana* verified that there was no significant increase in viral content in plants when DCL2 and DCL4 were interfered with the expression of only one of the proteins by RNAi, but there was a significant increase in viral content when both were interfered with at the same time (Katsarou et al., 2019). This result confirms that DCL2 and DCL4 are associated with antiviral defense as well as the functional redundancy between the two. In a sresearch by Wang (Wang et al., 2024) Chromosome-level genome annotation indicates that *N.benthamiana* retains single copies of *RDR6*, *DCL2*, *DCL3*, and AGO2 following subgenome loss, and possesses fewer *AGO1* and *AGO4* homologs compared with *Nicotiana tabacum*. Such reductions in RNA silencing-associated gene copy number may partially underlie the increased viral susceptibility of *N.benthamiana*.

## 3 DNA virus-host interactions studied in *N.benthamiana*

Geminiviruses are among the most serious viruses affecting plants globally, posing a serious threat to the safety of economic and food crops such as tobacco, tomato, and cassava in particular (Li et al., 2018**;** Chen et al., 2013**;** Allie et al., 2014). As the most abundant plant DNA virus, its interactions with plants involve both the same PTGS pathway as against RNA viruses and a variety of different mechanisms, and its complete infection cycle remains unelucidated (Kumar, 2019**;** Aguilar et al., 2020**;** Liu et al., 2021). The genomes of Geminiviruses may have either one or two single-stranded circular DNAs, and containing a single ssDNA type requires the presence of satellite DNAs, while containing two ssDNA types usually requires the presence of both DNA-A and DNA-B to trigger systemic infection, both of which are rolled-over replicating in the host (Aguilar et al., 2020**;** Briddon and Stanley, 2006). Among all plant viruses, DNA viruses are not as diverse as RNA viruses, and among the few plant DNA viruses, Geminiviruses are currently the most widely studied. Therefore, the section on *N.benthamiana* interactions with plant DNA viruses is reviewed using representative Geminiviruses as examples.

Plants share the same primary defense mechanism against DNA viruses and RNA viruses, both of which rely on RNA silencing to block post-transcriptional expression, so natural mutations in *N.benthamiana RDR1* also affect interactions with DNA viruses (Akhtar et al., 2011**;** Prakash et al., 2020). However, *RDR1* can be able to enhance the methylation of the viral genome against Geminiviruses thereby alleviating symptoms (Basu et al., 2018). At this time the properties of RDR1 as a defense protein help to reduce the abundance of viral transcripts in plants and increase the level of siRNAs (Basu et al., 2018). Loss of functional *NbRDR1* equates to loss of this mechanism, and thus *N.benthamiana* is more defective in defense against DNA viruses compared to defense against RNA viruses.

Geminiviruses generally require the presence of both DNA-A and DNA-B in the host to cause systemic infection. It has been well documented that, influenced by innate susceptibility, the presence of only a portion of the virus’ DNA-A in *N.benthamiana* is sufficient to cause infection (Saunders et al., 2002**;** Galvao et al., 2003**;** Wang et al., 2014). In the presence of both DNA-A and DNA-B of *Indian cassava mosaic virus from Singapore* (ICMV-SG), the infected leaves of Jatropha curcas show yellowish-green mosaic, curling, deformities, overall stunted development of infected plants as well as fewer or even completely sterile fruits, but DNA-A expressing ICMV-SG in *N.benthamiana* resulted in the above mentioned conditions (Wang et al., 2014). This result demonstrates that *N.benthamiana* has the potential to explore the respective functions of the two parts of the DNA of Geminiviruses.

Viruses also have their unique means of interfering with plant defenses when plants defend themselves against viruses. The ability to regulate DNA methylation has been reported in Geminiviruses-plant interactions (Gui et al., 2022**;** Guo et al., 2022). On the one hand, plant RDR1 inhibits viral replication by methylation modification of virus DNA as described above, and on the other hand, the virus can regulate demethylation to disrupt methylation-mediated defenses (Gui et al., 2022). *N.benthamiana* defects cause a loss of function in the former, and thus the latter demethylation is more active, which may also be the reason for the *N.benthamiana* effect on biolytic virus replication (Gui et al., 2022). *N.benthamiana* is susceptible to Geminiviruses. Geminiviruses can encode a variety of small proteins, *Tomato yellow leaf curl virus* (TYLCV) encodes RNA silencing inhibitory protein V3 (Gong et al., 2021), and the AV2 protein of *Tomato leaf curl Palampur virus* (ToLCPalV) enhances viral virulence and accelerates *N.benthamiana* systemic infection. *N.benthamiana* system necrosis and accumulation of reactive oxygen species (ROS) (Roshan et al., 2018). The current study of viral small proteins using *N.benthamiana* as a model is shown in **Table 1**.

**Table 1:** Current research on small viral proteins in *N. Benthamiana.*.

Small proteins	Virus	Function in *Nicotiana benthamiana*
Plant RNA viruses		
P0	*Cotton leafroll dwarf virus;* *Brassica yellows virus, etc.*	Mediates the degradation of AGO proteins and impairs the antiviral activity of NbRAF2.
P1	*Rice yellow mottle virus;* *Cocksfoot mottle virus, etc.*	P1 can inhibit the activity of intact RISC.
P19	*Tomato bushy stunt virus*	Binding to sRNA prevents it from entering RISC.
P20	*Cucumber necrosis virus*	Binding to sRNA prevents it from entering RISC.
P23	*Citrus tristeza virus*	P23 has a zinc-finger structure that binds to RNA to prevent RNA silencing.
P25	*Potato virus X*	P25 can degrade part of the AGO protein.
P38	*Turnip crinkle virus*	Interaction with AGO protein exerts the function of silencing inhibition
2b	*Cucumber mosaic cucumovirus*	2b can inhibit RDR6-mediated secondary viral siRNA production and can inhibit Slicer activity of AGO proteins.
Hc-Pro	*Barley stripe mosaic virus*	Inhibit siRNA/miRNA assembly into RISC.
Plant DNA viruses		
AC2	*Mungbean yellow mosaic virus; African cassava mosaic virus*	Deactivators for viral transcription and inhibitors for RNA silencing.
AV2	*Tomato leaf curl Palampur virus*	AV2 is essential for systemic movement of DNA-A, symptom severity, and viral DNA accumulation.
V3	*Tomato yellow leaf curl virus*	The V3 protein is localized to the Golgi apparatus and acts as an inhibitor of RNA silencing.

(Cascardo et al., 2015; Sun et al., 2018; Siddiqui et al., 2008a; Sarmiento et al., 2007; Giner et al., 2010; Qu and Morris, 2002; Lakatos et al., 2006; Hao et al., 2011; Ruiz-Ruiz et al., 2013; Flores et al., 2013; Siddiqui et al., 2008b; Aguilar et al., 2015; Chiu et al., 2010; Thomas et al., 2003; Lucy et al., 2000; Jiang et al., 2012; Yelina et al., 2002; Trinks et al., 2005; Roshan et al., 2018; Gong et al., 2021).

## 4 Viroid-host interactions in *N.benthamiana*

Viroids are a class of small ring-closed, single-stranded RNA molecules without a protein capsid envelope, whose genomes must interact directly with host components in order to replicate themselves and induce disease in the host (Flores et al., 2015). Compared to plant viruses we know little about the interactions between viroids agents and plants. Viroid species have been shown to trigger the RNA silencing pathway, but the role of the RNA silencing pathway in viroid infections is unclear (Li et al., 2021).

When a viroid infects a plant, the viroid nucleic acid is subjected to degradation by the DCL protein into a 21–24 nt viroid-derived smallRNA (vd-sRNA), which is then assembled into a RISC for specific cleavage of homologous host mRNAs (Ma and Zhang, 2018). Infection of *N.benthamiana* with *Potato* sp*indle tuber viroid* (PSTVd), the process accompanying the production of vd-sRNA by the above mechanism can trigger a series of molecular events leading to the emergence of macroscopic symptoms of viroid infections, which in *N.benthamiana* are manifested as leaf curling and developmental retardation (Navarro et al., 2021).

Plant-induced RNA silencing responses against viroids require the activities of both *RDR1* and *RDR6*. To compensate for the loss of endogenous *RDR1* function in *N.benthamiana*, *NtRDR1* from *N.tabacum* was overexpressed. Upon inoculation with PSTVd, plants expressing *NtRDR1* exhibited a marked reduction in viroid RNA accumulation (Li et al., 2021). This result demonstrates that the resistance of *N.benthamiana* to viroids is affected by the absence of NbRDR1 function, and that NbRDR6 plays an important role in the accumulation of viroid RNA and infection symptoms in *N.benthamiana* (Di Serio et al., 2010). When the *NbRDR6* gene is repressed, symptoms do not appear at the beginning of infection despite high levels of viroid RNA accumulation, demonstrating that viroid-induced symptoms in *N.benthamiana* are dependent on the activity of *NbRDR6* (Gómez et al., 2008), and that repression of *NbRDR6* facilitates the accumulation of viroid RNA (Adkar-Purushothama and Perreault, 2019).

Unlike the case of plants against Euvirus, DCL4 was mentioned as an antiviral-associated DCL protein in *N.benthamiana* against RNA virus infestation, so when DCL4 expression is inhibited it results in a large increase in viral content, but paradoxically DCL4 inhibition leads to a decrease in viroid content (Dadami et al., 2013**;** Katsarou et al., 2016). The above findings were confirmed in *N.benthamiana*, where the interaction between viroids and RNA silencing defied the prevailing antiviral mechanism, reaffirming the idea that viroids need to rely on RNA silencing in plant in order to accomplish substantial replication.

Viroid pathogenicity largely resembles endogenous microRNA-mediated regulation of plant growth and development, a process essential for viroid replication while also engaging host defense responses. Owing to its high susceptibility, *N.benthamiana* provides an effective system for dissecting viroid infection mechanisms and host resistance, although many aspects of these interactions remain to be elucidated.

## 5 Bacterial interactions and immune responses in *N.benthamiana*

Bacterial-plant interactions are highly complex, *N.benthamiana* tissues contain abundant extracellular hydrolytic and antimicrobial enzymes that limit bacterial colonization and can compromise bacterial integrity, thereby reducing virulence (Sueldo et al., 2023). These hydrolytic enzymes accumulate during the invasion process, so that the pathogenic bacteria need either a strong enough cell wall or secondary metabolites that inhibit hydrolytic enzyme activity in order to further infest the tissue and cause disease (van Loon et al., 2006).

Upon successful pathogen invasion, plants employ two major immune strategies: Pathogen-AssociatedMolecular Pattern (PAMP)-triggered immunity (PTI) and effector-triggered immunity (ETI). PTI relies on PRR-mediated recognition of PAMPs at the cell surface, whereas ETI involves direct or indirect recognition of pathogen avirulence (Avr) proteins by intracellular NBS-LRR resistance proteins. Both PTI and ETI activate the mitogen-activated protein kinases (MAPK) signaling cascades that contribute to enhanced disease resistance and localized hypersensitive responses (HR) (Jones and Dangl, 2006**;** Schwessinger and Ronald, 2012**;** Qian and Liu., 2014).

In the early stages of infestation without releasing effectors, Pattern Recognition Receptors (PRRs) on plant cell membranes specifically recognize pathogenic bacterial PAMPs to induce a defense response. Most of the plant PTI studies have centered around flagellar proteins (Felix et al., 1999**;** Chinchilla et al., 2006). Recognition of flagellar proteins of *Pseudomonas syringae* by *N.benthamiana* is dependent on a homolog of the *A.thaliana* AtFLS2, NbFLS2 protein, which specifically recognizes a conserved flg22 polypeptide on the bacterial flagellum to induce PTI. NbFLS2 silenced *N.benthamiana* will not respond to flagellin induction and the growth of various pathogens will be dramatically enhanced, confirming that NbFLS2 plays an important role in flagellar perception (Hann and Rathjen, 2007). Elongation factor thermo unstable (EF-Tu) is a specific PAMP, and when a pathogen comes into contact with plant cells, the EF-Tu receptor (EFR) specifically recognizes the pathogen to release EF-Tu N-terminal elf18, producing a pathogen defense response that is highly similar to the flagellin-induced defense response (Kunze et al., 2004). EF-Tu is unique in that its receptor, EFR, is only found in *A.thaliana* and other Brassicaceae (Kunze et al., 2004). *Agrobacterium* is currently the most widely used plant genetic transformation system (Horsch et al., 1985), an EF-Tu-containing pathogen, when infected with *A.thaliana*, induces a ROS burst, which accelerates ethylene synthesis and other drastic defense responses that limit transformation of *A.thaliana* by *Agrobacterium*. In contrast, *N.benthamiana*, which is not a member of the Brassicaceae, is not responsive to EF-Tu and is not limited by this immune response, which confers the ability to efficiently transform *N.benthamiana* T-DNA and transient transfection (Zipfel et al., 2006).

**Figure 2:**
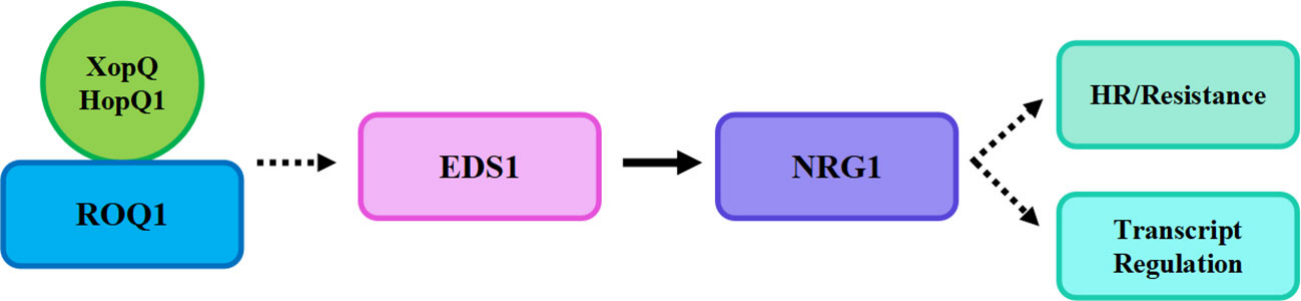
A simple model for TNL-mediated ETI signaling pathway. XopQ/HopQ1 forms a complex with ROQ1 and indirectly interacts with EDS1 to mediate NRG1-triggered transcriptional regulation or HR response.

The release and translocation of effectors of pathogenic bacteria into plant cells followed by specific recognition with the corresponding nucleotide-binding leucine-rich repeat (NLR) immune receptors leads to the activation of NLR proteins to induce ETI (Cui et al., 2015). In the ETI signaling pathway, NLR proteins require the cooperation of other molecules to be activated properly, and it has been confirmed by VIGS in *N.benthamiana* that the trio of NbSGT1, NbEDS1, and NbRAR1 functions to help NLR proteins form complexes with effectors (Peart et al., 2002**;** Liu et al., 2002b). Using *N.benthamiana* as a model to explore effectors XopQ and HopQ1 produced by *Xanthomonas* and *Pseudomonas*, respectively, Schultink found that a TIR-NLRs (TLRs) Recognition of XopQ 1 (Roq1) was able to specifically recognize XopQ and HopQ1 and confirmed that Roq1 is an indispensable component of Nicotiana genus resistance to *Xanthomonas* and *Pseudomonas*, and reported that the perception of XopQ and HopQ1 is dependent on EDS1 (Schultink et al., 2017). On this basis Qi further complemented the interaction of N requirement gene 1 (*NRG1*) with *EDS1* downstream of Roq1 and EDS1 to mediate ETI triggered by pathogenic XopQ and HopQ1, which ultimately leads to HR (Qi et al., 2018), a simple model of this signaling pathway is shown in **Figure 2**.

## 6 Oomycete-plant interactions analyzed using *N.benthamiana*

Potato late blight disease is a disease caused by *Phytophthora infestans* (*P. infestans*) infection, which is characterized by mildew on stems, leaves, and tubers that begins as yellowish-brown spots and rapidly expands to form dense white mold in humid climates, resulting in plant rot (Fry, 2008). The range of species infected by *P.infestans* is very narrow, generally occurring only in the Solanaceae, so *N.benthamiana*, which is related to cash crops such as tomato and potato, has become the most widely used model plant for the study of *P.infestans*-plant interactions (Kamoun et al., 1993**;** Kamoun et al., 1998).

INF1 is a class of elicitin proteins secreted by *P.infestans* that act as PAMPs in interactions with *N.benthamiana* (Kamoun et al., 1998). INF1 can localize to the extracellular compartment of *N.benthamiana* tissues, interacts with the receptor complex containing NbLRK1 on the surface of the plasma membrane and recognizes INF1, triggering downstream H_2_O_2_ production and HR signaling (Kanzaki et al., 2008). *In vitro* experiments have demonstrated that INF1 interacts with the subdomain VIb recognition of NbLRK1 intracellular kinase activity, and under natural conditions it is dependent on a number of proteins, including Hsp90, Hsp70, and SGT1, to successfully activate downstream cascade reactions (Kanzaki et al., 2008**;** Shibata et al., 2011). However, there are still many mysteries to be solved in the study of INF1. No research has been able to show how INF1 enters *N.benthamiana* cells and acts on NbLRK1 intracellular structures, and the composition of the NbLRK1 receptor complex remains unknown, although INF1-interacting membrane proteins have been validated *in vitro* (Kanzaki et al., 2008). On the other hand, *N.benthamiana* exportin 1 (NbXpo1) is required for INF1-induced defense responses, and in mammals and fungi exportin1 is thought to be a regulator of protein and RNA export through the nuclear pore, and NbXpo1-silenced *N.benthamiana* exhibits a slight growth defects, as well as inhibition of INF1-induced cell death, significantly reducing resistance to *P.infestans* (Mizuno et al., 2019). The heteropolyploid nature of *N.benthamiana* may underlie gene copy expansion, as *A.thaliana* contains two *Xpo1* genes (*AtXpo1a* and *AtXpo1b*), while *N.benthamiana* possesses four *Xpo1* homologs. A similar pattern is observed for *BAK1* and its homolog *NbSerk3*.(Blanvillain et al., 2008**;** Goodin et al., 2008). VIGS-mediated silencing of *NbSerk3A* and *NbSerk3B* in *N.benthamiana* significantly attenuated the hypersensitive response induced by purified INF1. These results indicate that *NbSerk3A* and *NbSerk3B* likely contribute to INF1-triggered cell death during Phytophthora infestans infection(Chaparro-Garcia et al., 2011).

## 7 Fungal interactions and host susceptibility factors in *N.benthamiana*

Fungal pathogens pose a serious threat to terrestrial plants, contributing to substantial yield losses of approximately 10-23% annually and further post-harvest losses of 10-20%. The high susceptibility and experimental accessibility of *N.benthamiana* make it a useful system for studying fungal pathogenesis and plant defense mechanisms (Stukenbrock and Gurr, 2023). Nowadays, fungi have become a great threat to food security, and the large-scale cultivation of monocultures in the field provides sufficient nutrient conditions and suitable breeding sites for fungi, and the use of antifungal agents under such conditions leads to the screening of resistant strains in a short period of time and their rapid multiplication (Fisher et al., 2018). Therefore, in this context, where the rate of fungal resistance formation is much faster than the rate of development of new antifungal agents, the study of plant-fungal interactions stands out.

*N.benthamiana* is an excellent model plant for studying the relationship between plants and fungal pathogens (Goodin et al., 2008). Fungal infection of plants begins with contact at the cell wall level, and the chitin-containing fungal cell wall is mechanically stronger than the tough and elastic bacterial cell wall. Plants do not contain chitin components, so chitin in fungal cell walls is considered a non-self component to activate plant immune responses. In the last decade of research, chitin is the most typical PAMP for fungi to cause PTI in plants (Kombrink et al., 2011). During fungal infection of Arabidopsis, *AtCERK1* and *AtLYKs*, members of the LysM-RLKs family, recognize chitin and activate downstream immune response pathways (Shinya et al., 2015). *NbCERK1* has been shown to be a chitin receptor and is involved in positively mediating the chitin signaling pathway (Gimenez-Ibanez et al., 2009), the defense mechanism of *N.benthamiana* is similar to that of Arabidopsis, which is recruited by AtLYK5 to form a receptor complex with AtCERK1 to bind chitin, whereas *N.benthamiana* binds chitin through the triple NbERK1/NbCERK1/NbLYK4 receptor complex, while NbERK1 with kinase activity dissociates from the complex and positively regulates the immune response in response to chitin induction, enhancing plant resistance (Pi et al., 2023).

**Figure 3:**
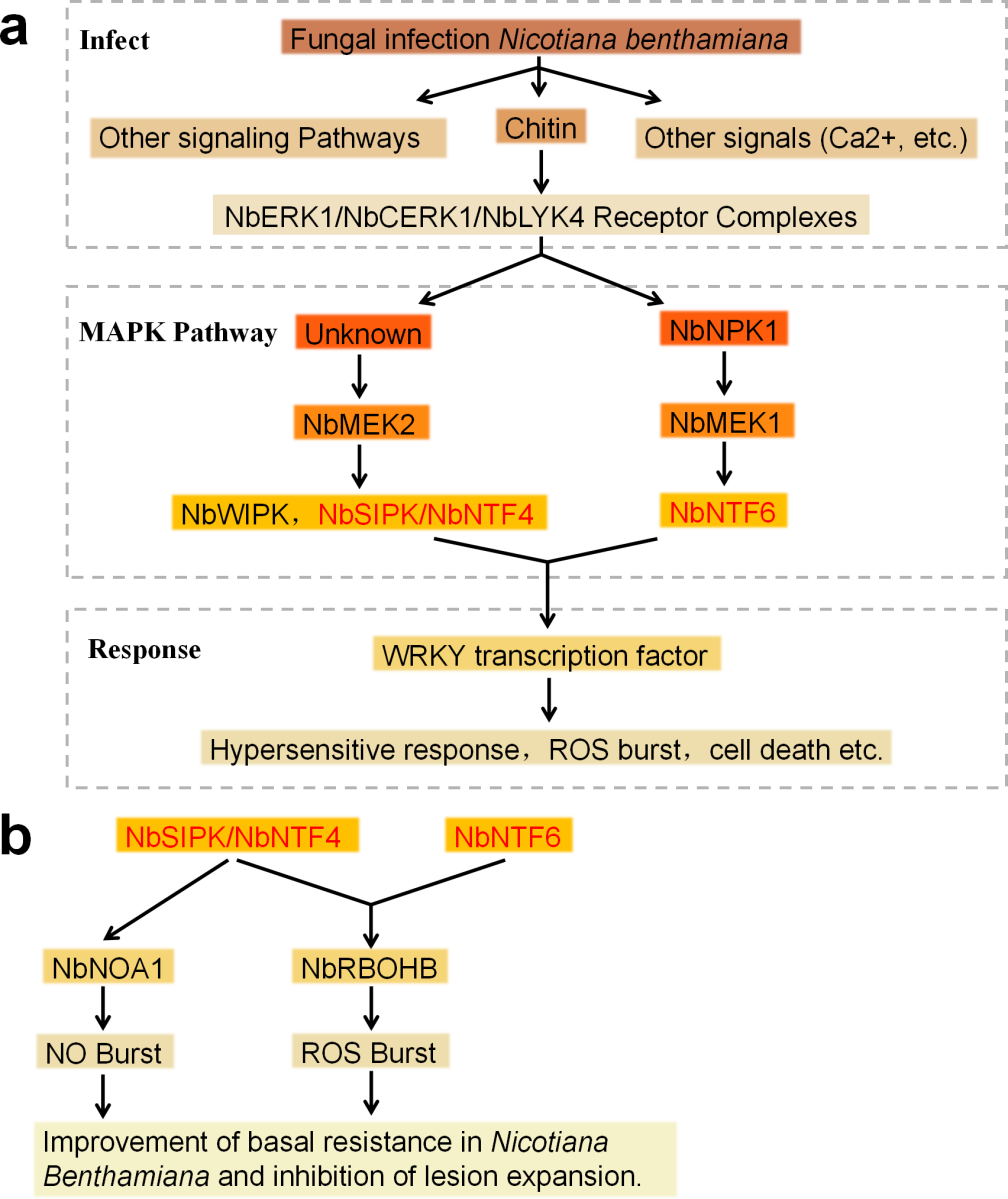
**(a)** A model for fungal infection signaling pathway in *N. Benthamiana.* **(b)** *N. Benthamiana* uses different signaling pathways to produce NO and ROS to improve basal resistance.

MAPK cascade reaction is a hallmark of defense signaling in plants such as Arabidopsis, tobacco, tomato, and *N.benthamiana*, and MAPK activity determines the effective level of basal plant resistance (Segonzac et al., 2011**;** Tanaka et al., 2009). Wound-inducible protein kinase (WIPK) and salicylic acid-inducible protein kinase, known as pathogen- responsive MAPKs, together comprise the MEK2-SIPK/WIPK cascade in *N.benthamiana* (Pedley and Martin, 2005). MEK2 is the common upstream kinase of SIPK and WIPK (Yang et al., 2001), and its active form, MEK2, has been shown to be the most effective kinase in *N.benthamiana*, and its active form, MEK2DD, induces phosphorylation of SIPK and WIPK, which in turn activates the downstream target WRKY transcription factor and elicits HR responses (Adachi et al., 2016). It has been demonstrated that phosphorylation of Positive Transcription Factor WRKY8 by SIPK and WIPK enhances DNA binding and transcriptional activity and increases expression of downstream defense genes in *N.benthamiana*. WRKY8 is also phosphorylated by NTF4, which is homologous to SIPK, and by NTF6 in the NPK1-MEK1-NTF6 cascade (Ishihama et al., 2011), as shown in **Figure 3a**. In *N.benthamiana* leaves, the MEK2-SIPK/NTF4 and MEK1-NTF6 cascades both regulate RBOHB, contributing to ROS bursts and restriction of lesion expansion; however, only MEK2-SIPK/NTF4 is required for effective ROS production and lesion containment. Furthermore, SIPK/NTF4 promotes nitric oxide (NO) accumulation by regulating Nitric Oxide-Associated 1 (*NOA1*), thereby enhancing basal resistance in *N.benthamiana* **Figure 3b** (Asai et al., 2008**;** Asai and Yoshioka, 2009).

## 8 Limitations and caveats of *N.benthamiana* as a model system

Despite its widespread use and unique experimental advantages, *N.benthamiana* is not a universal model for all aspects of plant-pathogen interactions. A critical evaluation of its limitations is essential for the appropriate interpretation of experimental results and for avoiding overgeneralization.

### 8.1 Genetic and physiological peculiarities of *N.benthamiana*

One well-recognized limitation of *N.benthamiana* is its genetic background, particularly the presence of mutations affecting RNA silencing pathways, such as deficiencies in RNA-dependent RNA polymerase activity.These features contribute to its exceptional susceptibility to a broad range of viruses, which has been instrumental for virology research. However, they may also exaggerate viral phenotypes compared with other plant species, potentially leading to conclusions that are not fully representative of natural host-virus interactions. Beyond the TMV and TBSV induced enhanced infection phenotypes discussed in chapter 2, comparative studies by Wylie et al. further suggest that laboratory accessions of *N.benthamiana* can display more severe disease outcomes than wild accessions upon infection with *Yellow tailflower mild mottle virus* (Wylie et al., 2015).

In addition, *N.benthamiana* exhibits physiological traits, including rapid growth and relatively weak basal immunity, that facilitate pathogen colonization. While these properties enhance experimental sensitivity, they may obscure subtle defense responses that are more readily detected in genetically robust systems such as *A.thaliana*.

### 8.2 Transient expression systems: strength and limitation

*Agrobacterium*-mediated transient expression is a cornerstone of *N.benthamiana* research, enabling rapid functional analysis of pathogen effectors and host immune components. For plant species lacking established stable transformation systems or characterized by low transformation efficiency, *N.benthamiana* transient expression system has become a central approach for functional gene analysis. This system has demonstrated strong utility in herbaceous plant research; for example, transient expression of the peanut resistance gene *AhRRS5* in *N.benthamiana* leaves robustly induced HR and significantly enhanced resistance to *Ralstonia solanacearum* (Zhang et al., 2017). This heterologous platform is equally effective for woody perennials. Overexpression of the apple LysM domain-containing protein gene *MdCERK1–2* in *N.benthamiana* activated defense responses, including H_2_O_2_ burst and accelerated lignin deposition, thereby conferring enhanced resistance to *Alternaria alternata* (Chen et al., 2020).

Moreover, the versatility of this system transcends phylogenetic boundaries, enabling functional studies in evolutionarily distant lineages as well as metabolically complex medicinal plants. Immune signaling proteins originating from mosses, such as *Physcomitrium patens*, retain detectable biological activity when transiently expressed in *N.benthamiana*, highlighting functional conservation across deep evolutionary distances (Bressendorff et al., 2016). In addition, for species characterized by complex secondary metabolic networks, including *Salvia miltiorrhiza* and *Artemisia annua*, the *N.benthamiana* transient expression system provides an efficient heterologous platform for functional characterization of key enzymes involved in diterpenoid and sesquiterpenoid biosynthetic pathways (Fang et al., 2017**;** Kanagarajan et al., 2012).

However, transient overexpression can result in non-physiological protein levels, ectopic localization, or artificial activation of immune responses. Such effects may complicate the interpretation of phenotypes, particularly when assessing weak or quantitative interactions. Furthermore, transient assays typically bypass endogenous transcriptional and post-transcriptional regulatory mechanisms, which may be critical for fine-tuning immune signaling. Consequently, conclusions drawn from transient expression experiments should ideally be validated using stable transgenic lines or complementary systems.

### 8.3 Limited translational relevance for crop species

Although *N.benthamiana* serves as a powerful discovery platform, not all findings can be directly extrapolated to crop plants. Immune receptors, signaling components, and downstream defense outputs often display lineage-specific diversification. Kourelis and van der Hoorn demonstrated pronounced differences in R gene modes of action among plant species and emphasized that immune mechanisms characterized in model plants cannot be assumed to be effective in crop species (Kourelis and van der Hoorn, 2018). This limitation underscores the importance of integrating *N.benthamiana*-based discoveries with follow-up studies in relevant crop systems, particularly when the ultimate goal is translational or applied research (Jones et al., 2016).

### 8.4 Incomplete representation of complex immune interactions

Plant immunity involves highly coordinated interactions between PTI, ETI, hormone signaling, and developmental processes. While *N.benthamiana* excels at dissecting individual components of these networks, it may not fully capture the spatial, temporal, and cell-type-specific complexity observed under natural infection conditions. This is especially relevant for interactions involving long-term colonization, tissue-specific responses, or systemic signaling.

Emerging approaches such as single-cell transcriptomics, spatial omics, and quantitative imaging may help address some of these limitations when combined with *N.benthamiana*-based assays.

## 9 Future perspectives on the application of *N.benthamiana*

*N.benthamiana* has been widely adopted as a model for plant-pathogen interaction studies, and insights gained from this system are broadly applicable to other Solanaceous species, including food crops such as tobacco, tomato, chili pepper, goji berry, and potato, as well as ornamental plants like *Datura stramonium*. Elucidating these interactions facilitates the improvement of resistance to biotic stresses while supporting plant growth and development.

### 9.1 Virus-induced gene silencing as a rapid platform for gene function analysis in *N.benthamiana*

*N.benthamiana* has become an indispensable experimental material in plant research. The VIGS technology, which was developed based on its susceptibility to viruses, has been optimized to form a fast and efficient system over the past 20 years and has been widely used in gene function studies (Burch-Smith et al., 2004**;** Ramegowda et al., 2014). A variety of plant viruses have been developed as VIGS vectors, such as PVX and TRV (Ruiz et al., 1998**;** Liu et al., 2002a), etc. Virus-induced gene silencing (VIGS) is an RNAi-based technique that induces systemic gene silencing in young *N.benthamiana* plants, enabling functional analysis of genes that are difficult to study by knockout due to lethality. In contrast to gene knockout, RNAi-based approaches are less likely to trigger compensatory upregulation of homologous genes (Ma et al., 2019). The only regret is that the phenotype caused by VIGS cannot be passed on to future generations, and can only be observed in the injected plants, and the phenotype caused by VIGS gradually disappears around 4 weeks after injection. Nearly 50 plant species have been shown to be suitable for VIGS, but *N.benthamiana* remains the core member with the widest applicability (Lange et al., 2013).

### 9.2 *Agrobacterium*-mediated transient expression for protein localization and pathway validation

*Agrobacterium*-mediated transient transformation provides access to target proteins in a short period of time and, as a eukaryote with a post-translational modification pathway, is more precise than prokaryotic expression systems (Gelvin, 2005). Currently, the most widespread application of *Agrobacterium*-mediated transient expression in *N.benthamiana* is subcellular localization. Proteins are typically expressed under the control of the CaMV 35S promoter and fused to fluorescent tags such as GFP or mCherry (Sheludko, 2008), reaching peak expression within 2–3 days after infiltration and remaining detectable for up to 5 days. This technique has become a routine tool for subcellular localization studies and provides a rapid heterologous platform for assessing the functional conservation of signaling pathways across plant species. In the future, combining *Agrobacterium*-mediated transient expression with advanced imaging and functional genomics approaches may further enhance its application in dissecting plant signaling pathways.

### 9.3 *N.benthamiana* as a plant-based bioreactor: advantages and limitations

*N.benthamiana* is widely used as a plant-based bioreactor in synthetic biology due to its high efficiency in *Agrobacterium*-mediated transient expression (Sheludko et al., 2007). This system bypasses the need for stable transgenic lines and complex pathway reconstruction, enabling rapid and scalable heterologous protein production through simple leaf infiltration. In addition, the robust environmental adaptability and low input requirements of *N.benthamiana* contribute to reduced production costs.

However, compared with microbial fermentation systems, plant-based bioreactors have several limitations. The longer growth cycle of plants, the destructive nature of product extraction, and the release of interfering cellular components during tissue disruption complicate downstream purification and reduce yield. Moreover, large-scale application of *N.benthamiana* requires substantial natural resources, including land, light, and water, in contrast to the compact and reusable infrastructure of microbial fermenters. (Wu et al., 2021).

## 10 Conclusion

*N.benthamiana* has emerged as an indispensable model system for dissecting plant-pathogen interactions, owing to its unique evolutionary background, genetic features, and exceptional experimental tractability. Its heteropolyploid genome, high susceptibility to diverse pathogens, and amenability to rapid genetic manipulation have enabled mechanistic insights into antiviral RNA silencing, immune signaling networks, and pathogen virulence strategies that are difficult to achieve in many other plant systems.

**Figure 4:**
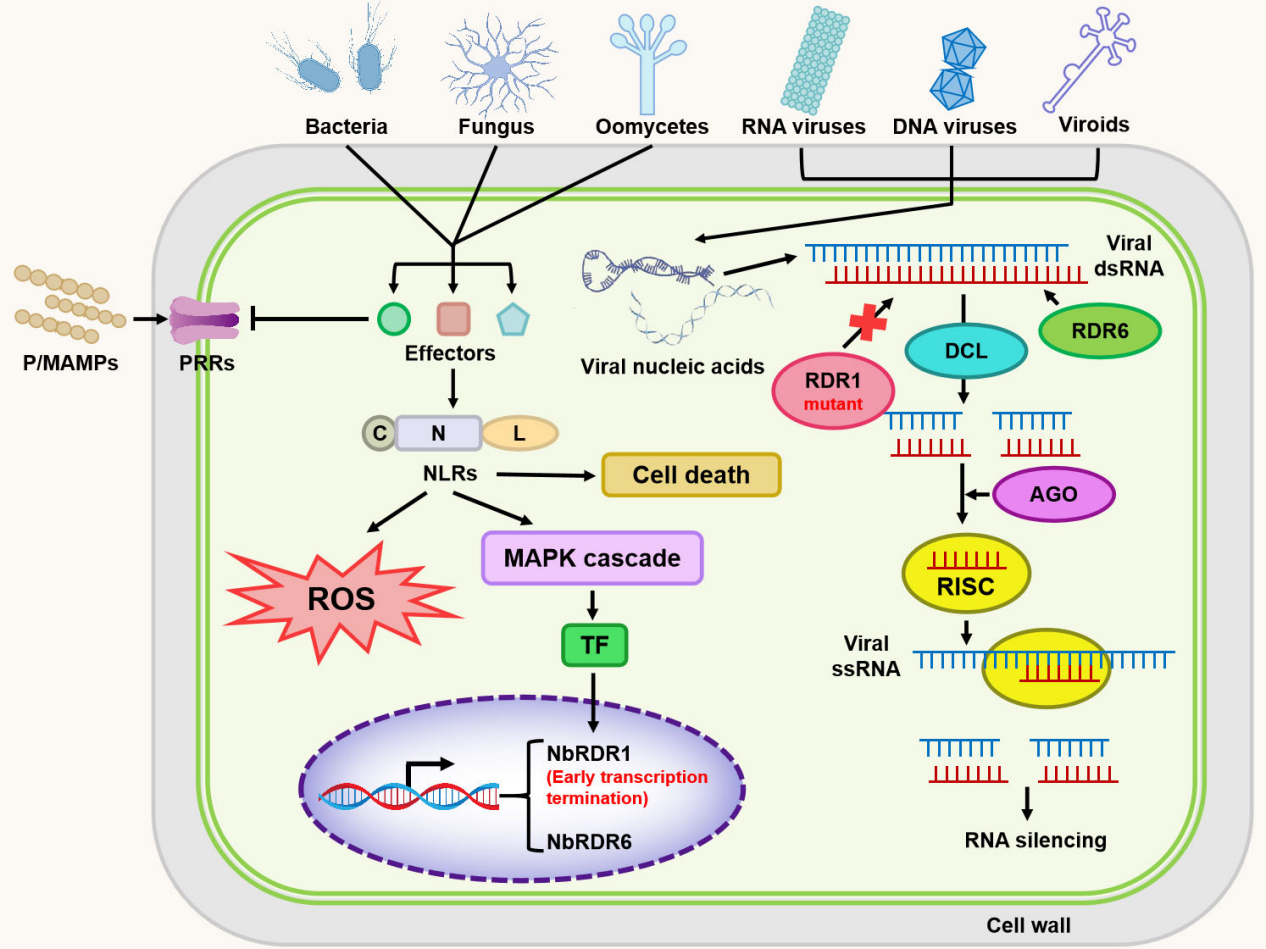
Mechanism of *N. Benthamiana* resistance to different pathogenic microorganisms. Due to early transcriptional termination, *NbRDR1* is nonfunctional, and viral RNA amplification and RNA silencing are therefore predominantly mediated by *NbRDR6*. Viral and viroid-derived nucleic acids are processed by DCL proteins into small RNAs, which are subsequently loaded onto AGO proteins to form RISCs, thereby mediating post-transcriptional gene silencing. In parallel, pathogen-associated molecular patterns or effector proteins from bacteria, fungi, and oomycetes activate MAPK cascades through PRR-mediated PTI and NLR-mediated ETI, leading to ROS production and the induction of cell death responses.

Studies in *N.benthamiana* have been particularly instrumental in elucidating RNA silencing-based antiviral defense, including the coordinated functions of RDRs, DCLs, and AGOs, as well as their complex regulatory relationships. Beyond viral systems, *N.benthamiana* has proven to be a powerful platform for investigating plant interactions with DNA viruses, viroids, bacteria, fungi, and oomycetes, revealing conserved and divergent features of PTI, ETI, MAPK signaling, and effector recognition across plant lineages (**Figure 4**). These findings highlight the value of *N.benthamiana*a as a unifying experimental framework for comparative analyses of plant immunity.

At the same time, the limitations of *N.benthamiana* as a model system must be carefully considered. Genetic peculiarities affecting RNA silencing, reliance on transient expression systems, and incomplete representation of complex immune dynamics underscore the need for cautious interpretation and validation in additional plant species. Rather than diminishing its utility, these caveats define the appropriate scope of *N.benthamiana* as a discovery-driven platform.

Looking forward, the integration of *N.benthamiana*-based approaches with emerging technologies will further enhance its capacity to uncover dynamic and context-dependent immune mechanisms. These technologies include single-cell and spatial omics, advanced imaging, and synthetic biology. Moreover, coupling discoveries made in *N.benthamiana* with translational studies in crop plants will be essential for converting fundamental insights into practical strategies for improving disease resistance and sustainable agriculture.

In summary, *N.benthamiana* will continue to serve as a cornerstone model for plant-pathogen interaction research, bridging fundamental mechanistic studies and applied plant science, and remaining central to future advances in plant immunity and biotechnology.
